# Supplementary material for: Spinal cord extracts of amyotrophic lateral sclerosis spread TDP-43 pathology in cerebral organoids
Source: PLoS Genet. 2023 Feb 6;19(2):e1010606. doi: 10.1371/journal.pgen.1010606 (PMC9934440; doi:10.1371/journal.pgen.1010606)
Supplement: S1 Table — Human iPSCs used in this study were generated from peripheral blood mononuclear cells (PBMCs) of a healthy control (AJC001 line; OrgCtrl) or sporadic ALS-FTLD patient (TD17 line; OrgALS). N.A. indicates “not assessed”. (PDF) [file pgen.1010606.s001.pdf]

S1 Table

Cell line	Diagnosis	Genetics	Material source	Gender	Age	Ethnicity
AJC001 (OrgCtrl)	Healthy control	N.A.	PBMCs	Male	37	Caucasian
TD17 (OrgALS)	Sporadic ALS-FTLD	Negative for *C9orf72* gene and ALS panel	PBMCs	Female	65	Caucasian
