# Supplementary material for: Spinal cord extracts of amyotrophic lateral sclerosis spread TDP-43 pathology in cerebral organoids
Source: PLoS Genet. 2023 Feb 6;19(2):e1010606. doi: 10.1371/journal.pgen.1010606 (PMC9934440; doi:10.1371/journal.pgen.1010606)
Supplement: S2 Table — Postmortem frozen spinal cords tissue specimens were collected from the Montreal Neurological Institute-Hospital (MNI), the Douglas-Bell Canada Brain Bank (DBCBB) and the London Neurodegenerative Diseases Brain Bank (LNDBB). N.A. indicates “not assessed”. (PDF) [file pgen.1010606.s002.pdf]

S2 Table

Case number	Diagnosis	*C9orf72* gene	Region	Gender	Age at onset	Age at death	Post-mortem delay (hours)	Source
Control 1	Cerebral hemorrhage	N.A.	Cervical	Female	65	65	unknown	MNI/DBCBB
Control 2	-	-	Cervical	Female	-	72	41	LNDBB
Control 3	-	-	Cervical	Male	-	63	23	LNDBB
Patient 1	Sporadic ALS	Normal	Cervical	Male	46	50	12	MNI/DBCBB
Patient 2	Sporadic ALS	Normal	Cervical	Female	59	62	22	MNI/DBCBB
Patient 3	Sporadic ALS	Expanded	Cervical	Male	unknown	51	10	MNI/DBCBB
Patient 4	Sporadic ALS	Normal	Cervical	Female	unknown	58	31	MNI/DBCBB
Patient 5	Sporadic ALS	Normal	Cervical	Male	78	79	27	MNI/DBCBB
