# Supplementary material for: Spinal cord extracts of amyotrophic lateral sclerosis spread TDP-43 pathology in cerebral organoids
Source: PLoS Genet. 2023 Feb 6;19(2):e1010606. doi: 10.1371/journal.pgen.1010606 (PMC9934440; doi:10.1371/journal.pgen.1010606)
Supplement: S3 Table — IF, immunofluorescence; IHC, immunohistochemistry; WB, Western blot; DB, dot blot; N.A. indicates "not assessed". (PDF) [file pgen.1010606.s003.pdf]

S3 Table

Antibody (clone)	Source (catalog number)	Host	IF	IHC	WB/DB
CTIP2 (25B6)	Abcam (ab18465)	rat	1:500	N.A.	N.A.
PAX6 (Poly19013)	Bio Legend (901301)	rabbit	1:100	N.A.	N.A.
SOX2 (9-9-3)	Abcam (ab79351)	mouse	1:200	N.A.	N.A.
TUJ1 (Poly18020)	Bio Legend (802001)	rabbit	1:5000	N.A.	N.A.
TUJ1	Abcam (ab41489)	chicken	1:1000	N.A.	N.A.
phospho TDP-43 (pS409/410)	Cosmo Bio (TIP-PTD-M01)	mouse	1:1000	N.A.	1:2000
phospho TDP-43 (pS409/410)	Cosmo Bio (TIP-PTD-P02)	rabbit	1:1000	N.A.	N.A.
phospho TDP-43 (pS409)	Cosmo Bio (TIP-PTD-P03)	rabbit	N.A.	1:1000	N.A.
TDP-43	Proteintech (10782-2-AP)	rabbit	1:200	N.A.	N.A.
GFAP	Invitrogen (PA5-16291)	rabbit	1:200	N.A.	1:2000
GR repeat	Proteintech (23978-1-AP)	rabbit	1:100	N.A.	1:1000
cleaved caspase-3 (E83-77)	Abcam (ab32042)	rabbit	1:200	N.A.	1:500
γH2AX (pS139)	Novus Biologicals (NB100-384)	rabbit	1:1000	N.A.	1:10000
anti-rat Alexa Fluor 488-conjugated	Invitrogen	goat	1:1000	N.A.	N.A.
anti-rabbit Alexa Fluor 488-conjugated	Invitrogen	goat	1:1000	N.A.	N.A.
anti-rabbit Alexa Fluor 555-conjugated	Invitrogen	goat	1:1000	N.A.	N.A.
anti-mouse Alexa Fluor 488-conjugated	Invitrogen	goat	1:1000	N.A.	N.A.
anti-mouse Alexa Fluor 555-conjugated	Invitrogen	goat	1:1000	N.A.	N.A.
anti-chicken Alexa Fluor 488-conjugated	Invitrogen	goat	1:1000	N.A.	N.A.
anti-rabbit peroxidase-conjugated	Invitrogen	donkey	N.A.	N.A.	1:10000
anti-mouse peroxidase-conjugated	Invitrogen	donkey	N.A.	N.A.	1:10000
