# Supplementary figures and images for: Spinal cord extracts of amyotrophic lateral sclerosis spread TDP-43 pathology in cerebral organoids
Source: PLoS Genet. 2023 Feb 6;19(2):e1010606. doi: 10.1371/journal.pgen.1010606 (PMC9934440; doi:10.1371/journal.pgen.1010606)

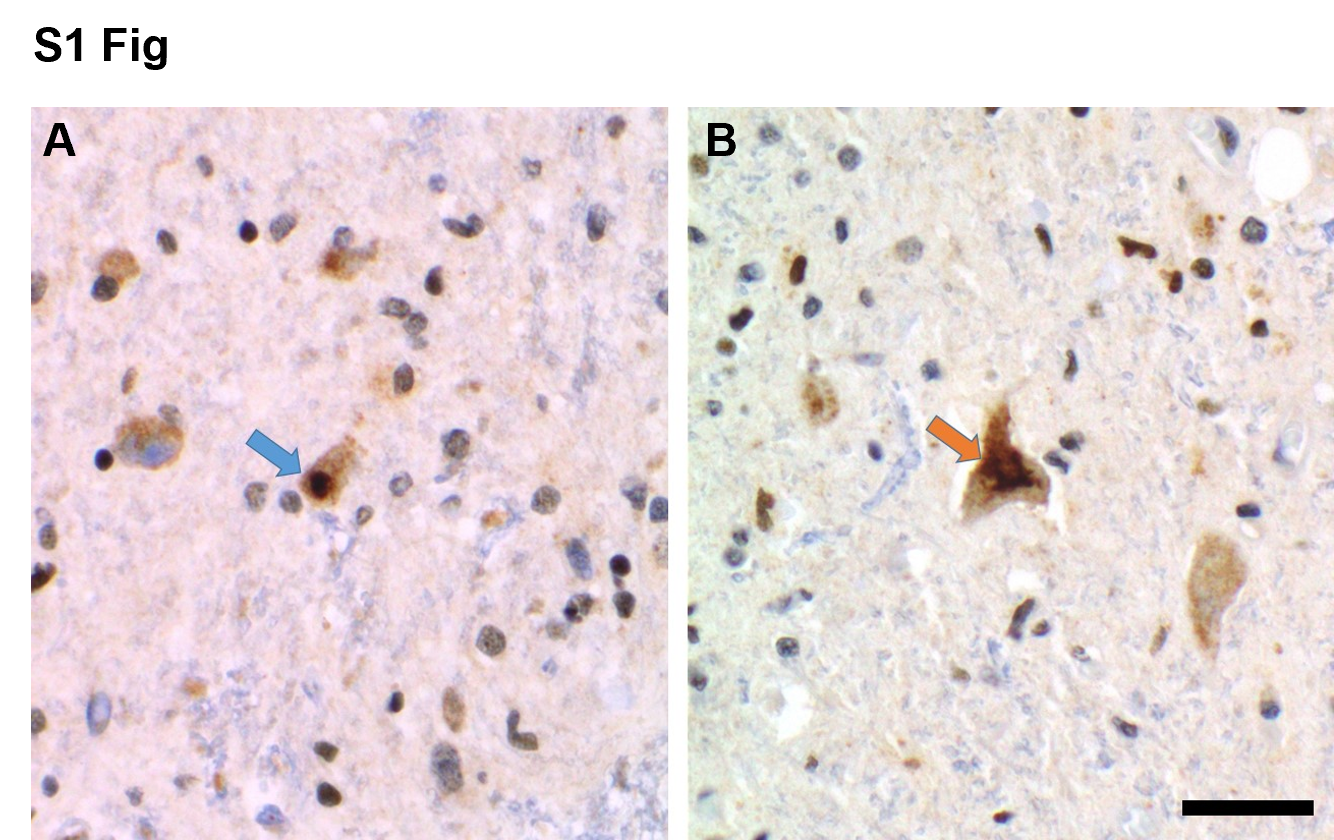

Supplement: S1 Fig — (A and B) Immunohistochemistry of anterior horn spinal cords stained by TDP-43 antibody. The blue arrow shows punctate intraneuronal inclusion (A) and the orange arrow shows skein-like intraneuronal inclusion (B). Scale bar = 50 μm. (TIF) [file pgen.1010606.s004.tif]

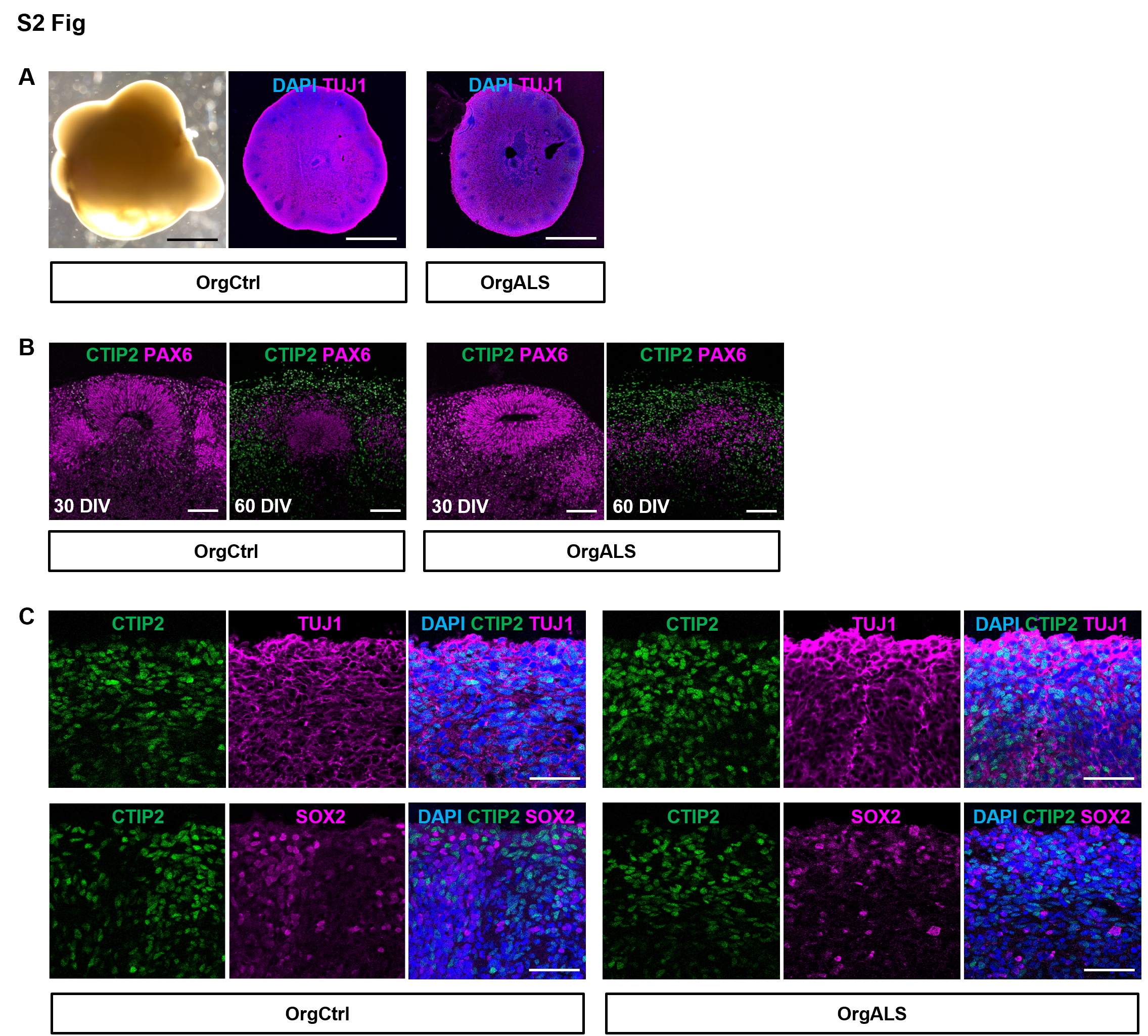

Supplement: S2 Fig — (A) Representative bright-field (left) and immunofluorescence images (right) of cerebral organoids differentiated from OrgCtrl or OrgALS iPSCs line at 60 DIV. Scale bars = 1 mm. (B) Double-label immunofluorescence images of CTIP2 and PAX6 staining OrgCtrl or OrgALS cerebral organoids at 30 DIV and day 60 DIV. Scale bars = 100 μm. (C) Double-label immunofluorescence images of CTIP2 and TUJ1 (upper panels) or SOX2 (lower panels) staining OrgCtrl or OrgALS cerebral organoids at 60 DIV. Sections were counterstained with DAPI to label the nuclei. Scale bars = 50 μm. (TIF) [file pgen.1010606.s005.tif]

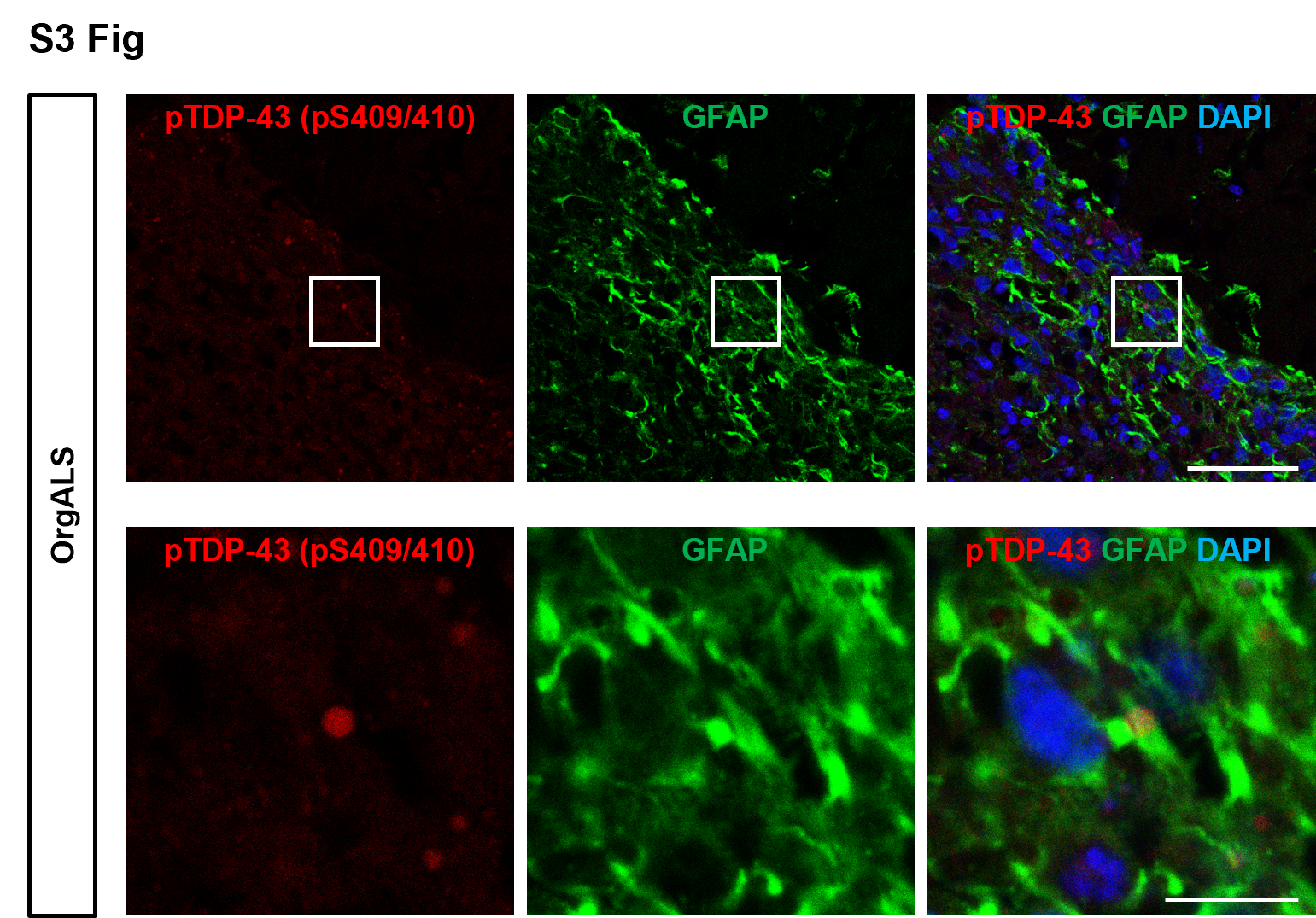

Supplement: S3 Fig — Immunofluorescence images of OrgALS cerebral organoid double-labelled with pTDP-43 and GFAP at 8 weeks post injection of protein extracts from ALS (patient 5). The lower panels are higher magnifications of the white-line boxes in the upper panels. Scale bars = 50 μm (upper panel) and 10 μm (lower panel). (TIF) [file pgen.1010606.s006.tif]

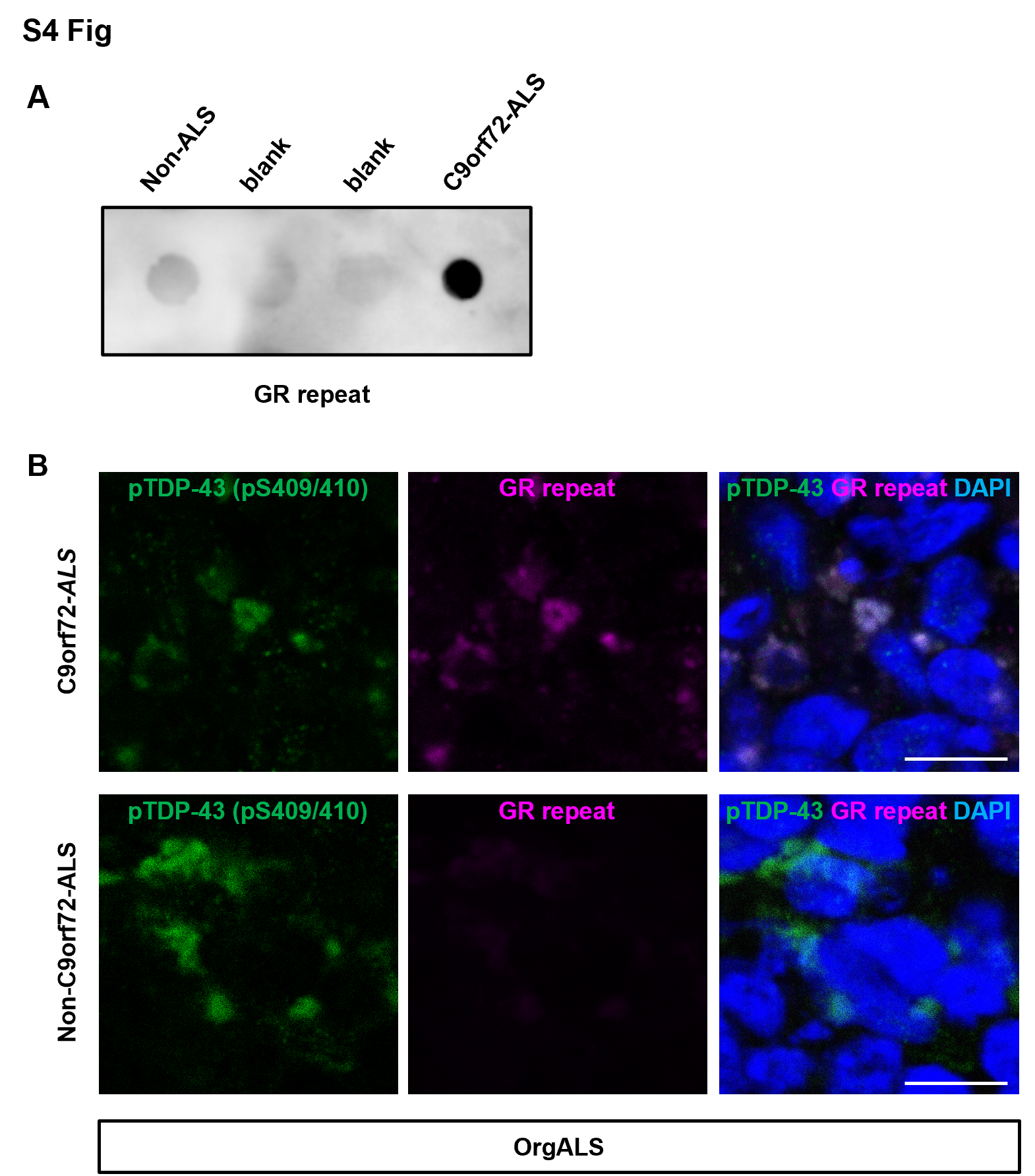

Supplement: S4 Fig — (A) Dot blot analysis of sarkosyl-insoluble protein extracts from non-ALS control (control 1) and C9orf72-ALS spinal cords (patient 3), immunoblotted with GR repeat antibody. (B) Immunofluorescence images of OrgALS cerebral organoids double-labelled with pTDP-43 and GR repeat proteins at 8 weeks post injection of protein extracts from C9orf72-ALS (patient 3) (upper panels) or non-C9orf72-ALS (patient 4) (lower panels). Scale bars = 10 μm. (TIF) [file pgen.1010606.s007.tif]
